# Decomposing County-Level Working-Age Mortality Trends in the United States Between 1999–2001 and 2015–2017

**DOI:** 10.1007/s40980-021-00095-6

**Published:** 2021-08-24

**Authors:** Nick Graetz, Irma T. Elo

**Affiliations:** 1 Population Studies Center, University of Pennsylvania, 3718 Locust Walk, Philadelphia, PA 19104, USA; 2 Department of Sociology, Population Aging Research Center, University of Pennsylvania, Philadelphia, USA

**Keywords:** Working-age mortality, Geographic inequality, Spatial statistics, County-level characteristics, Decomposition

## Abstract

Studies have documented significant geographic divergence in U.S. mortality in recent decades. However, few studies have examined the extent to which county-level trends in mortality can be explained by national, state, and metropolitan-level trends, and which county-specific factors contribute to remaining variation. Combining vital statistics data on deaths and Census data with time-varying county-level contextual characteristics, we use a spatially explicit Bayesian hierarchical model to analyze the associations between working-age mortality, state, metropolitan status and county-level socioeconomic conditions, family characteristics, labor market conditions, health behaviors, and population characteristics between 2000 and 2017. Additionally, we employ a Shapley decomposition to illustrate the additive contributions of each changing county-level characteristic to the observed mortality change in U.S. counties between 1999–2001 and 2015–2017 over and above national, state, and metropolitan–nonmetropolitan mortality trends. Mortality trends varied by state and metropolitan status as did the contribution of county-level characteristics. Metropolitan status predicted more of the county-level variance in mortality than state of residence. Of the county-level characteristics, changes in percent college-graduates, smoking prevalence and the percent of foreign-born population contributed to a decline in all-cause mortality over this period, whereas increasing levels of poverty, unemployment, and single-parent families and declines manufacturing employment slowed down these improvements, and in many nonmetropolitan areas were large enough to overpower the positive contributions of the protective factors.

## 1 Introduction

Geography, reflecting the social and political context of one’s area of residence, has long been recognized as an important factor influencing an individual’s exposures to health-related risks, access to health services, and educational and economic opportunities over a person’s life course (Chetty et al., 2014; Hillman, 2016; Krieger et al., 2005; Wen et al., 2003). In the United States, recent studies have documented large geographic inequalities in health and mortality across Census regions and divisions (Elo et al., 2019; Fenelon, 2013), commuting zones (Chetty et al., 2016b), states (Montez et al., 2019; Montez et al., 2017; Montez et al., 2016; Wilmoth et al., 2011; Woolf & Schoomaker, 2019), metropolitan and nonmetropolitan areas (Cossman et al., 2010; Eberhardt & Pamuk, 2004; Elo et al., 2019; James, 2014; James et al., 2018; Singh & Siahpush, 2014), and counties (Dwyer-Lindgren et al., 2017; Wang et al., 2013). Not only have these studies documented large geographic inequalities in health and mortality at a point in time but growing geographic inequalities over time that contribute to the low ranking of U.S. life expectancy internationally (Dobis et al., 2020; Montez et al., 2020; Wilmoth et al., 2011; Woolf & Schoomaker, 2019). Factors that have been hypothesized to contribute to these disparities include state-level policies that affect health and well-being (Montez et al., 2016, 2017, 2020), geographic variation in socioeconomic inequalities and economic opportunities (Chetty et al., 2016a; Dwyer-Lindgren et al., 2017; Monnat, 2018), distribution of health care resources and access to care (James & Cossman, 2017; Monnat, 2019), population composition (Spencer et al., 2018), and health-related behaviors (Dwyer-Lindgren et al., 2017; Tencza et al., 2014).

This paper contributes to the above literature by examining to what extent the variation in county-level mortality can be attributed to national, state, and metropolitan–nonmetropolitan-level mortality trends and which changing county-level characteristics, including socioeconomic, labor market and family characteristics, health behaviors and population composition, contribute to the remaining variation. Due to the instability of small-area mortality estimates at the county-level and the presence of spatial autocorrelation across counties (Dwyer-Lindgren et al., 2016), we employ a spatially explicit Bayesian hierarchical model to estimate the associations between mortality rates, states, metropolitan status, and our county-level characteristics measured in 2000 and 2017. We use a Shapley decomposition to examine how changes in these county-level characteristics contribute to county-level mortality trends. By aggregating the contributions of these characteristics by state and metropolitan status, we examine how their contributions to mortality trends have varied by state and metropolitan-nonmetropolitan status. We focus on mortality trends in young adulthood and midlife (ages 25–64). Mortality trends at these ages, in contrast to older ages, have been particularly adverse and have contributed to the decline in U.S. life expectancy since 2014 (Elo et al., 2019; National Academies of Sciences, Engineering, and Medicine 2021; Vierboom et al., 2019; Woolf & Schoomaker, 2019).

## 2 Background

Regional differences in U.S. mortality are longstanding with the South having had consistently higher mortality and lower life expectancy than the rest of the United States (James et al., 2018; Woolf & Schoomaker, 2019). Within the South, life expectancy is lowest in the South-Central division having increased by only 0.9 years between 2000 and 2016 from 74.5 to 75.4 years. This pattern contrasts to the Northeast and the Pacific divisions, which experienced larger increases in life expectancy, rising from 78.2 years in 2000 to 80.2 years in 2016 in the Northeast and from 78.1 years to 80.9 years in the Pacific (Woolf & Schoomaker, 2019). In addition to this regional variation in U.S. mortality, there is considerable state-level heterogeneity within regions. In 2016, the state-level life expectancy gap in the South between the lowest (Mississippi—74.7 years) and highest (Florida—79.5 years) life expectancy was 4.8 years. The respective figures were 2.7 years in the Northeast, 3.9 years in the Midwest, and 3.6 years in the continental West (Woolf & Schoomaker, 2019).^1^

Others have drawn attention to divergent health and mortality trends by metropolitan status. Mortality in nonmetropolitan areas has exceeded that in metropolitan areas nationally since the mid- to late-1980s (Cosby et al., 2008; Cossman et al., 2010) and exhibited variation within the nonmetropolitan spectrum (James, 2014) and across U.S. regions. Elo et al. (2019) documented declining life expectancy among non-Hispanic white women between 1990–1992 and 2014–2016 in nonmetropolitan areas of Appalachia, East South Central and West South-Central regions, but varying increases in life expectancy in nonmetropolitan areas in other regions and among nonmetropolitan men in all regions. In contrast, non-Hispanic white male and female life expectancy increased in metropolitan areas in all regions. James and Cossman (2017) document higher excess mortality in rural areas compared to urban areas for whites since the mid- to late-1980s and for blacks since the late 1990s.

Within states and sub-regions, studies have further identified clusters of high and low mortality across the United States. Murray et al. (2006) documented a life expectancy gap of 15.4 years for Asian versus high-risk urban black males and a 12.8-year gap for Asian versus low-income Southern rural black females in 1997–2001 (Murray et al., 2006). Chetty et al. (2016a, 2016b) highlighted divergent trends in life expectancy at age 40 by income and commuting zone across the United States using individual-level data from the Internal Revenue Service and Social Security Administration between 2001 and 2014. Over this period, life expectancy increased by 2.34 years for men and 2.91 years for women in the top 5% of the income distribution but by only 0.32 years for men and 0.04 years for women in the bottom 5%. In the bottom income quartile, life expectancy varied substantially among commuting zones with a gap of 4.5 years between the lowest and highest life expectancy. The lowest life expectancies were concentrated in parts of the South, southern parts of the Midwest, and scattered throughout the West (Chetty et al., 2016b) (Fig. 5). Studies examining county-level mortality trends further highlight variation not only across regions and states but also across counties within states (Figs. 5 and 6), with the highest concentrations of high mortality counties in the southern states, especially in the Mississippi Delta and Appalachia, in counties with large Native American populations, and in a scattering of counties throughout the rest of the country (Wang et al., 2013). More recently, Vierboom et al. (2019) examined geographic variation in life expectancy at birth between 1990 and 2016 across 40 geographic areas defined by region and metropolitan status and documented growing divergence in life expectancy by metropolitan status and region of the country (see also Elo et al., 2019). Subsequently, Vierboom and Preston (2020) used county-level mortality data to examine geographic trends in life expectancy at age 65 between 2000 and 2016. They further documented increasing geographic inequalities in life expectancy with largest increases occurring in large metropolitan areas and on the East and the West Coasts (Vierboom & Preston, 2020). The recent National Academies of Science, Engineering and Medicine report on working-age mortality trends (ages 25–64) similarly highlighted widening geographic divergence in mortality by metropolitan status, state, and region of the country (National Academies of Sciences, Engineering, and Medicine 2021).

### 2.1 Proposed Explanations for Geographic Variation in Mortality

Theoretical frameworks for elucidating geographic variation in mortality emphasize multiple layers of influence ranging from societal and community-level factors to individual-level characteristics, with increasing emphasis placed on more distal social, economic and policy contexts that shape health outcomes through multiple interacting mechanisms, including but not limited to employment opportunities and working conditions, and health-related knowledge and health behaviors (Braveman et al., 2011; Montez et al., 2016, 2017, 2020; National Academies of Sciences, Engineering, and Medicine, 2021; Solar & Irwin, 2010). At the national level, medical discoveries, health care policies, and economic trends can impact all regions of the country, although their effect can vary by state and local context. For example, the implementation of the Affordable Care Act (ACA), passed in 2010 with the goal of expanding health insurance coverage, controlling costs, and improving the delivery of health care, has been uneven across the U.S., resulting in expanded health insurance coverage for the previously uninsured everywhere, but by a varying degree depending on the state. Griffith et al. (2017), for example, found that the income gap in health insurance coverage between households with incomes above $75,000 and below $25,000 narrowed by 14% (from 31 to 17%) in states that expanded Medicaid coverage under the ACA compared to 8% (from 36 to 28%) in states that did not expand Medicaid during the period 2011–2015 (Griffith et al., 2017).

Several studies have documented an association between mortality and state-level variation in policies, economic and social context. In a study of state-level variation in women’s mortality at ages 45–89, Montez et al. (2016), for example, found that state-level contextual characteristics (economic environment, social cohesion, socio-political orientation, physical infrastructure, and tobacco environment) together accounted for 62% of the mortality variation among states, with economic conditions and social cohesion being the most important. In a related study, Montez and et al. (2020) linked changes in life expectancy between 1970 and 2014 to changes in state policies on a conservative-liberal continuum. In states that enacted more conservative policies life expectancies were more likely to decline or improve more slowly than in states with more liberal policies on tobacco, immigration, civil rights, private labor, environment, gun control, LGBT rights and abortion, with some variation in the strength of the association between men and women and specific policy domain. Furthermore, educational disparities in mortality grew between 1985–1998 and 1999–2011 in some states but not all, especially in the South and the Midwest, suggesting that state-level context can also shape educational mortality inequalities (Montez et al., 2019; see also Montez et al., 2017 for state-level educational disparities in disability). In addition to state-level policies, economic and social context, the prevalence of health-related behaviors, such as smoking and obesity predict state-level variation in all cause and cause-specific mortality (Patel et al., 2014; Tencza et al., 2014).

At the county level, socioeconomic characteristics and behavioral risk factors are also associated with county-level mortality variation (Dobis et al., 2020; Dwyer-Lindgren et al., 2017), and area-level population composition plays a role in geographic variation in mortality. For example, Dwyer-Lindgren et al., (2017, Abstract) reported that “[s]socioeconomic and race/ethnicity factors, behavioral and metabolic risk factors, and health care factors explained 60%, 74%, and 27% of county-level variation in life expectancy” in 2009 (Dwyer-Lindgren et al., 2017). Monnat (2018) finds that economic distress is associated with drug-related mortality, net of opioid supply factors (Monnat, 2018, 2019). Across commuting zones and among US counties, a higher proportion of the population that is foreign-born is also associated with lower mortality and higher life expectancy (Chetty et al., 2016a, 2016b; Dobis et al., 2020), whereas higher percentages of black and rural residents are associated with higher mortality (Cosby et al., 2019).

Our study adds to this literature by describing differential trends in age-standardized crude death rates (ASCDRs) in young adulthood and midlife (25–64) across all U.S. counties between 2000 and 2017 and by examining the degree to which shifting county-level profiles of socioeconomic, labor force and family characteristics, health behaviors, and population composition explain heterogeneous mortality trends by state and metropolitan–nonmetropolitan continuum. We focus on these characteristics as they have been identified as important predictors in a cross-sectional analysis of county-level variation in mortality and have a potential to explain trends in geographic inequalities in mortality. We assembled a database of these time-varying contextual characteristics at the county-year-level in 2000 and 2017.

## 3 Data and Methods

### 3.1 Mortality and Population Data

We use vital statistics data on deaths combined with estimates of the population at risk prepared by the Census Bureau in 1999–2001 and 2015–2017. Mortality estimates are pooled across three adjacent years to avoid issues related to very small numbers of deaths in certain county-age-years. We use restricted microdata files on deaths by age, sex, county, and year for the continental United States obtained from the National Center for Health Statistics (NCHS) under a data user agreement. We use public-use Census bridged-race population estimates by age, sex, county, and year. We focus the analyses on ages 25–64, the ages at which recent mortality trends have been most adverse (Case & Deaton, 2015, 2020; Elo et al., 2019; National Academies of Sciences, Engineering, and Medicine, 2021).

### 3.2 County-Level Characteristics

Our county-level contextual characteristics reflect changes in the county’s socioeconomic characteristics (percent college graduates, percent under the federal poverty threshold), labor force profile (percent employment in manufacturing, percent unemployed), family characteristics (percent of families with children under 18 and a single householder) health behaviors and risk factors (prevalence of smoking and obesity), and population composition (percent of the total population at ages 25–64, percent of the total population that is foreign-born) between 2000 and 2017. Education is an important predictor of mortality at the individual-level with mortality disparities by educational attainment increasing over time (Geronimus et al., 2019; Hendi, 2017; Montez et al., 2019). Aggregate area-level measures of educational attainment are also associated with mortality and health outcomes (Chetty, et al., , 2016a, 2016b; Monnat, 2018). Area-level economic deprivation, measured by percent of the population in poverty, captures a different dimension of the county’s socioeconomic profile.

One of the contributors to changing county-level economic profile is changes in its labor market conditions. One of the sectors of the economy that has been particularly affected by macro-level economic trends and foreign competition is manufacturing with the decline in manufacturing jobs being uneven across the country with long lasting consequences for employment and wages (Charles et al., 2019). The loss of well-paying manufacturing jobs with few alternative employment opportunities can devastate communities, lead to out-migration, family disruption, a loss of a sense of community, and increased unemployment. The impact of trade policies and a decline in manufacturing jobs has been associated with opioid use and “deaths of despair” (Charles et al., 2019; Pierce & Schott, 2016; Venkataramani et al., 2020). Changes in county or state level unemployment rate in turn have been linked to an increase in deaths from opioids (Hollingsworth et al., 2017) and to suicide mortality (Phillips & Nugent, 2014; Ruhm, 2000). Drug-related and suicide mortality are key contributors to the increase in working-age mortality between 2000 and 2017 (National Academies of Sciences, Engineering, and Medicine, 2021). Economic disruption can also lead to lower marriage rates or family disintegration reducing a key form of social support. We include percent single parent families to capture county-level family dynamics.

Smoking and obesity are two key health behaviors affecting trends in U.S. mortality (Preston & Stokes, 2011; Preston et al., 2014) and are the two health behaviors included in this analysis. The final two measures capture county-level population composition. The first is the percent of the total population that is working-age to capture both in- and out-migration of the working-age population over the period. The second captures changes in the foreign-born population. The percent foreign-born has been shown to be a significant predictor of life expectancy at age 40 among U.S. commuting zones (Chetty et al., 2016a, 2016b), and the foreign-born population in New York City accounted for all of the life expectancy advantage in New York City compared to the United States in 2010 (Preston & Elo, 2014).

To capture geographic variation in mortality, we include the state where the county is located and the county’s metropolitan–nonmetropolitan (hereafter metropolitan) status. To classify counties by metropolitan status, we used a modified version of the codes developed by the Economic Research Service, USDA and are available from the National Center for Health Statistics (Ingram & Franco, 2014). We distinguish four types of areas: large central metros, their suburbs (large fringe metros), medium/small metros, and nonmetro areas. To maintain consistency over time, we use the counties’ metropolitan category as of 2013. We use the combination of state and metropolitan category together with the county-level characteristics to assess the relative contributions of each to the widening geographic inequalities in mortality between 2000 and 2017.

### 3.3 Statistical Analysis

We begin by defining and exploring spatial autocorrelation across working-age mortality rates at the county-level. We calculate a global Moran’s I using all county age-standardized (25–64) overall mortality rates for 2000 and 2017 to assess spatial autocorrelation in our data. The Moran’s I statistic in Eq. (1) is a common measure of spatial autocorrelation, i.e., the strength of the correlation between observations nearer in space compared to those that are further apart (Li et al., 2007).


(1) $$I=\frac{N}{W}\frac{\sum_{i}\sum_{j}w_{ij}(x_{i}-\bar{x})(x_{j}-\bar{x})}{\sum_{i}(x_{i}-\bar{x}{)}^{2}}$$

*N* is the number of spatial units indexed by *i* and *j*, where *i* is each specific county, *j* is every other county, *x* is the observed county-level mortality rate, $\bar{x}$ is the mean mortality rate across all counties, *w* is a matrix of spatial weights, and *W* is the sum of all spatial weights. This statistic is dependent on assumptions about the structure of the spatial weight matrix. For this analysis, we use a Queens matrix which defines “neighbors” as those counties sharing a boundary. Additionally, we calculate a test examining local indicators of spatial autocorrelation (LISA) over the entire dataset using the same spatial weights matrix (Anselin, 2010). This approach decomposes the global Moran’s I statistic to local pockets of spatial autocorrelation, and tests whether these clusters are significantly different from what could be expected given the same observed data randomly distributed across space (weighting with county populations). This exploratory test can be useful in locating clustered observations with significant leverage on the global spatial autocorrelation, as well as identifying local pockets of potential spatial non-stationarity.

To examine the associations between mortality and our county-level and geographic predictors, we fit Bayesian generalized linear models with a binomial likelihood and logit link-function to estimate mortality rates (*m*_i,y,a,s_) in county *i*, year *y*, sex *s*, and five-year age group *a* (Eqs. 2, 3) (Allison, 2014). This specification for small area mortality estimation is described in Clark et al. (2013) and has been widely adopted in similar settings (Clark et al., 2013; Golding et al., 2017; Wakefield et al., 2019). We compared this to the more conventional Negative-Binomial model and found results to be virtually identical. As described by Clark et al. (2013) and Wakefield et al. (2019), there are reasons to prefer the Binomial logit-link model when fitting more complicated random effects models for mortality rate data (e.g., spatial autocorrelation models), including greater numerical stability.

All models include discrete year, 2000 or 2017, (*Y*), sex (*S*), five-year age-groups (*A*), and a spatial random effect, *γ_i_*, following the Besag-York-Mollie (BYM) model (Eq. 4). The BYM model for the spatial random effect includes a spatially structured variance parameter following the Besag distribution (*τ*_*Besag*_) and a spatially independent variance parameter (*τ_iid_*). The spatial weights matrix was constructed as above using a nearest-neighbors approach following the Queens convention. All models were fit in a Bayesian framework with uninformative priors. The posterior distributions were fit using computationally efficient and accurate approximations in R-INLA (integrated nested Laplace approximation) (Rue et al., 2014).

Model 1 controls age, sex, and year. We then introduce metropolitan categories (***M_i_***) by year interactions in Model 2, state (***T_i_***) by year interactions in Model 3, and all time-varying county-level predictors (***X_i,y_***) in Model 4 in addition to both the metropolitan and state by year interactions:

(2) $$D_{i,y,a,s}|m_{i,y,a,s},N_{i,y,a,s}∼Binomial(m_{i,y,a,s},N_{i,y,a,s})$$

(3) $$logit\left(m_{i,y,a,s}\right)=\beta_{0}+\beta_{1}X_{i,y}+\beta_{2}Y*\left(T_{i}+M_{i}\right)+\beta_{3}A+\beta_{4}S+\gamma_{i}+\varepsilon_{i,y,a,s}$$

(4) $$\gamma_{i}∼BYM(\tau_{Besag},\tau_{Normal})$$

We used the deviance information criterion (DIC) to compare model performance. The DIC is a hierarchical modeling generalization of the more commonly used Bayesian information criterion (BIC). As with BIC, a smaller DIC is preferable in comparison and penalizes models for fit as well as the number of effective parameters.

We use Shapley decomposition to decompose the observed change in country-level mortality rates to the contributions of (1) shifting compositions in each time-varying county-level predictor, (2) the national trend, (3) states trends, (4) metropolitan trends, and (5) unexplained residual change (Fortin et al., 2011; Madden, 2012; Wang et al., 2014). Shapley decomposition is a permutation-based multivariate decomposition method with a game theory foundation that is typically used to decompose a multivariate *R*^2^ value to the contributions from each separate predictor variable (Grömping, 2006). Shapley decomposition can also be used as a permutation-based approach to Blinder-Oaxaca decomposition for non-linear models where the goal is to explain the difference in the means of a dependent variable between two groups by decomposing the gap into the part that is due to (1) differences in the mean values of the independent variable within the groups, and (2) group differences in the effects of the independent variable (Wang et al., 2014). In our case, we are decomposing the observed difference in mortality rates between 2000 and 2017 for each county. Specifically, to assess the contribution of each time-varying independent variable in our model to the change in mortality rates between 2000 and 2017, we constructed all scenarios in which each explanatory variable took on values from either 2000 or 2017. To compute the contribution of any one explanatory factor to the change in mortality between 2000 and 2017, we assessed the conditional expectation in each pair of scenarios in which that explanatory variable changed but all others maintained either their 2000 or 2017 values. The average of this expectation across all scenarios was the contribution of a given explanatory variable to total change in observed mortality.

Shapley decomposition will give an identical answer to Blinder-Oaxaca decomposition for OLS models, but the benefit of the permutation-based Shapley approach is being able to perform the same decomposition task with any generalized linear model (i.e., non-normal likelihoods, complex random effects). However, in these settings it is not an exact solution to the Blinder-Oaxaca decomposition, but an approximation; for example, by averaging over non-linearities across the logit distribution when we average over all scenarios of a given changing predictor. We test the concordance between the observed mortality changes from 2000 to 2017 and the sum of the decomposed changes attributable to each independent factor. We find error introduced by the Shapley approximation to be negligible (*R*^2^ = 0.99, mean absolute error = 3.25 per 100,000). For additional details on the Shapley decomposition approach, see Appendix Section 1.

We focus only on changes in the composition of county-level characteristics over time, rather than on changes in year-specific coefficients. We tested year-specific models and found that the coefficients did not change substantially between 2000 and 2017. In contrast, the levels of the county-level covariates not only varied by state and metropolitan status, but they also shifted markedly over time (Table 1).

## 4 Results

### 4.1 Descriptive Results

**Fig. 1:**
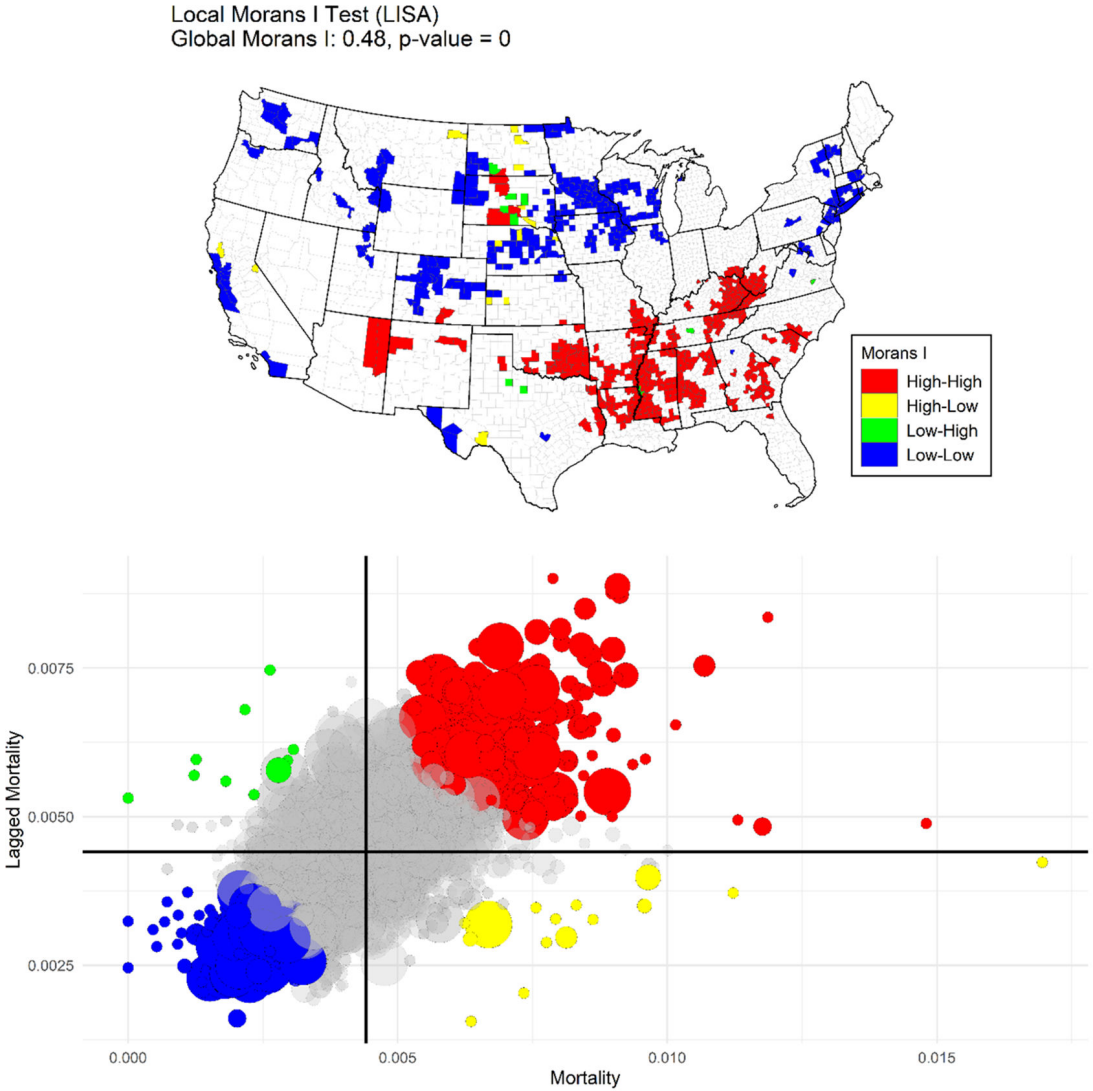
Global and LISA tests for spatial autocorrelation across county-level age-standardized all-cause U.S. mortality rates, ages 25–64, 2015–2017. *Note*: The scatter plot shows each county’s mortality rate compared to the rates of surrounding counties defined by the Queens spatial matrix. Dot size corresponds to county population. Counties are highlighted with a significant LISA test for positive spatial autocorrelation (*p* < 0.05). Red indicates high-mortality counties surrounded by similarly high-mortality counties and blue indicates low-mortality counties surrounded by similarly low-mortality counties. Green and yellow indicate spatial “outliers”; counties with significant negative spatial autocorrelation (high-mortality surrounded by low-mortality in yellow; low-mortality surrounded by high-mortality in green). The map visualizes the location these counties across the United States.

Figure 1 displays the global (Moran’s I) and local indicators of spatial autocorrelation (LISA) tests across age-standardized death rates at ages 25–64 in 2015–2017. Both tests reveal significant autocorrelation in the death rates across space (Global Moran’s I = 0.48). High mortality clusters are in Appalachia, the Mississippi Delta and in several southern states. Pockets of low mortality are found in the plain states of North and South Dakota, in parts of the Midwest, some Mountain states and as well as in parts of the Middle Atlantic and the Pacific regions. In many parts of the country, county-level mortality rates are not significantly different from the clustering we might expect if rates were distributed randomly across counties.

**Fig. 2:**
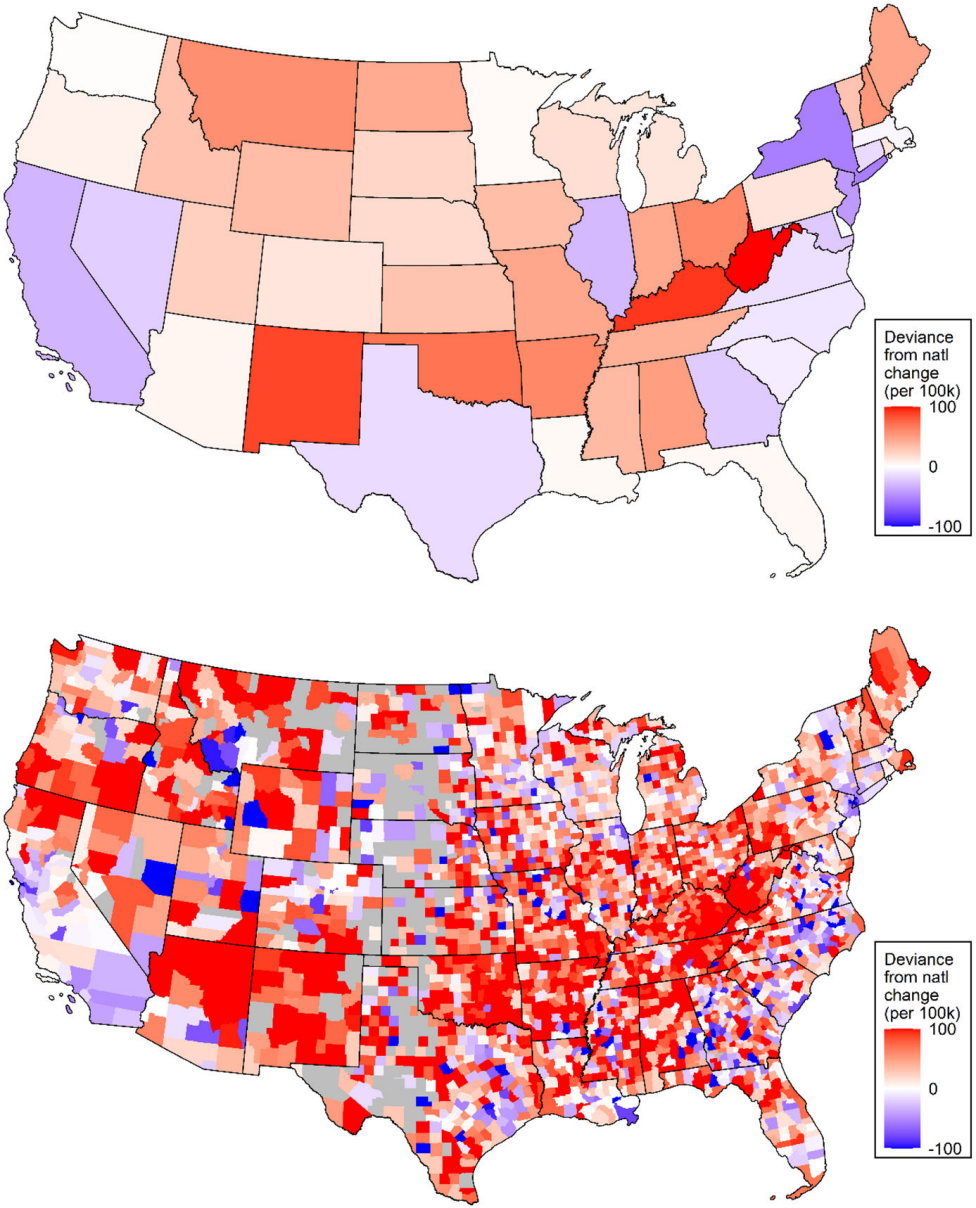
Deviance from national change in age-standardized all-cause U.S. mortality rate between 1999–2001 and 2015–2017 by state and county, ages 25–64. *Note*: While all counties are included in the analysis, here we suppress counties in grey with populations below 2000 to avoid visual artifacts resulting from large variability across very sparsely populated counties.

**Fig. 3:**
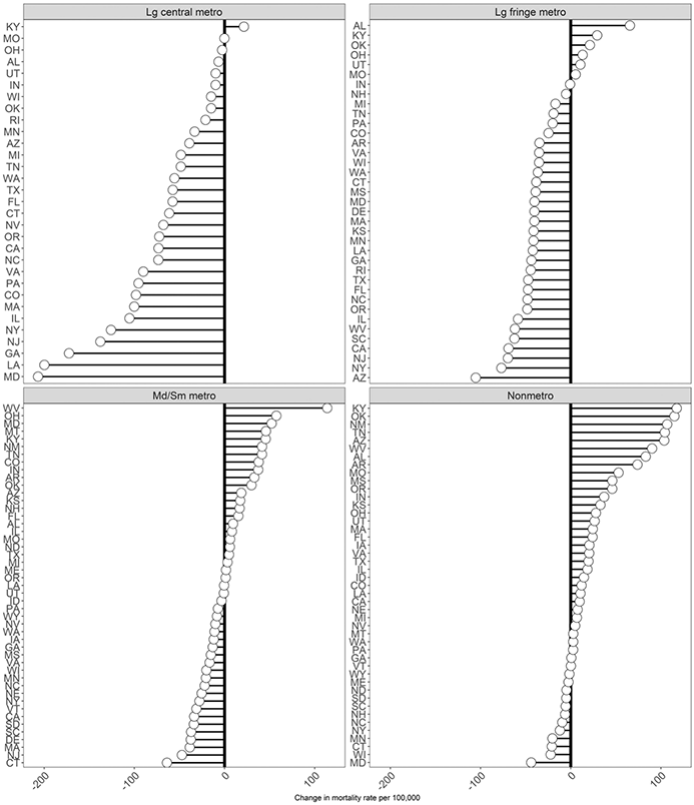
Change in age-standardized U.S. all-cause mortality rates between 1999–2001 and 2015–2017 by metropolitan–nonmetropolitan category and state, ages 25–64

Figure 2 illustrates deviance from the national change in the ASCDR at ages 25–64 (1999–2001 to 2015–2017) at the state and county levels. Mortality trends were especially adverse in West Virginia, Kentucky, and New Mexico. Several Mid-Western, Southern, Mountain and some New England states also experienced mortality trends that were more unfavorable than the national average. In contrast, states located on the East and West coasts as well as some parts of Texas and Illinois experienced more favorable trends in working-age mortality than the national average. At the same time, mortality change exhibited considerable variation among counties in all states. Furthermore, as shown in Fig. 3, which presents the change in ASCDR by metropolitan category and state, *declines* in mortality were most pronounced in large central metropolitan areas, with mortality increasing only in Kentucky. Similarly, in 31 of the 37 states with large fringe metropolitan areas mortality also declined (Fig. 3). In contrast, mortality trends were much less favorable in small/medium metropolitan and nonmetropolitan areas, with mortality declining in small/medium metros in only 24 of 47 states and in only 12 of 45 states in nonmetropolitan areas. At the same time, there was also variation by state within each of the metropolitan categories. It is clear from Figs. 1, 2 and 3 that US mortality trends between 1999–2001 and 2015–2017 have been quite heterogenous by state, metropolitan category, and county.

### 4.2 Results of Regression Analyses

**Fig. 4:**
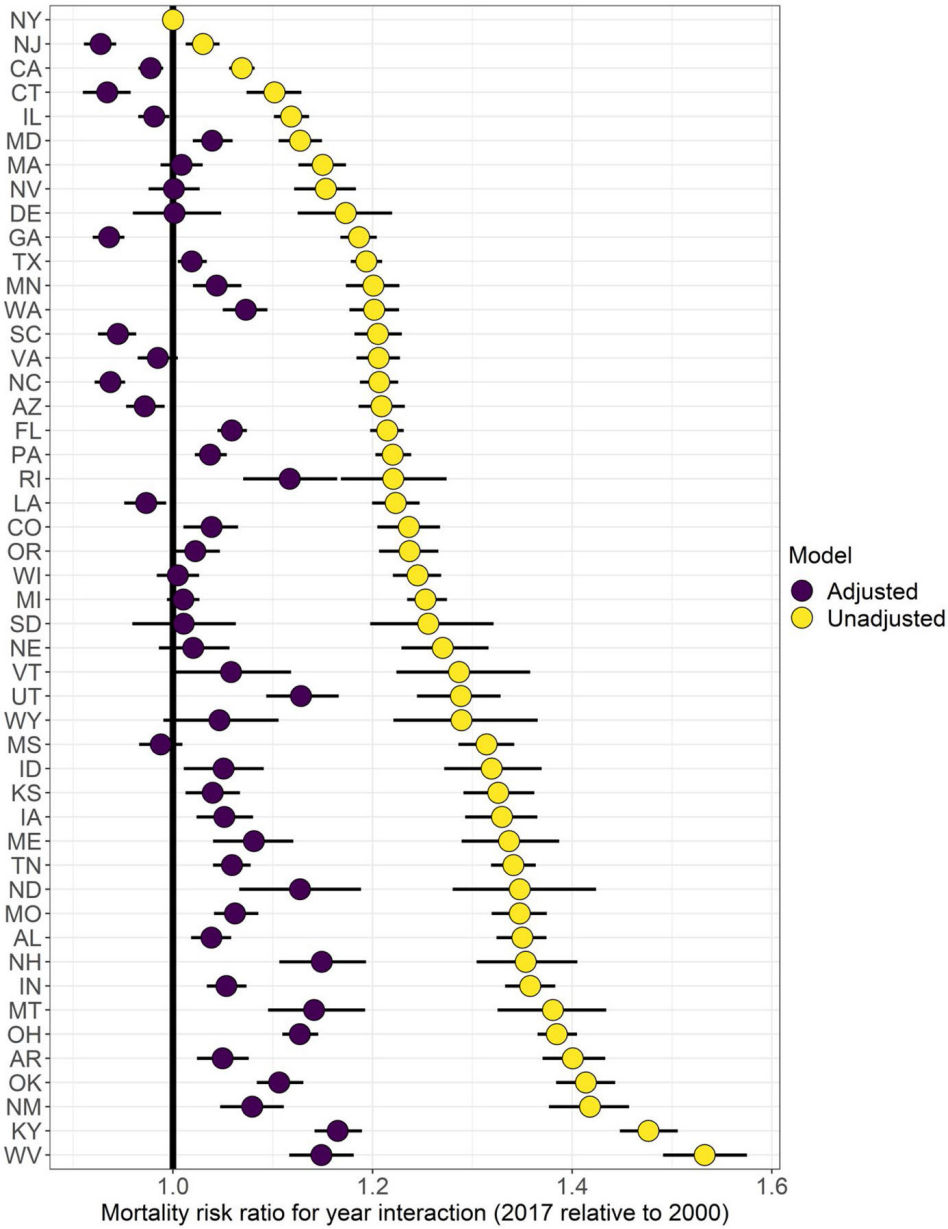
Estimated coefficients (mortality risk ratios) for a change in U.S. all-cause mortality rate between 1999–2001 and 2015–2017 by state unadjusted (Model 3) and adjusted (Model 4) for metropolitan status and county-level characteristics (reference = New York), ages 25–64.

Table 2 presents the results from the regression analyses. All estimates for the county-level explanatory factors are presented as mortality risk ratios and in terms of standard deviations for each characteristic (calculated across all values from 2000 and 2017). In the baseline Model 1, we control only for year, age, and sex. In Models 2 and 3 we introduce metropolitan category by year (reference = large central metro, 1999–2001) and state by year (reference = New York, 2000) interactions, respectively. Model 2 shows that in 1999–2001, all metropolitan categories were associated with lower county-level mortality rates compared to large central metros (though only the risk ratio for large fringe metros had a Bayesian credible interval that did not overlap with 1). However, mortality rates in all metropolitan categories increased dramatically between 1999–2001 and 2015–2017 relative to large central metros; for example, mortality rates were 1.30 (95% credible interval: 1.29–1.31) times higher in nonmetros in 2015–2017 than in 1999–2001 relative to large central metros. For clarity we do not present all state by year coefficients from Model 3 in this table, but these are illustrated in Fig. 4. Relative to New York, mortality increased in all states between 2000 and 2017, with largest increases in West Virginia and Kentucky and smallest increases in New Jersey and California. Model 2 had a lower DIC than Model 3, suggesting that only including the metropolitan category by year interaction terms produces a better model fit than Model 3, which included all state by year interaction terms.

We include all county-level characteristics in Model 4, which as expected produces the lowest DIC. In terms of socioeconomic, family and labor force characteristics of counties, high proportions living in poverty (1.04; 1.03–1.04) and higher proportion of single parent families (1.07; 1.06–1.07) were associated with higher mortality rates while high proportions of college attainment were associated with lower mortality rates (0.90; 0.89–0.91). A higher percentage of the labor force in manufacturing (0.98; 0.97–0.99) was associated with lower mortality, whereas a higher percentage of the population unemployed was associated with higher mortality (1.06; 1.05–1.07). The two features of the population composition included in our models were associated with lower mortality rates: a higher percentage of the total population in working-ages (0.95; 0.94–0.96) and a higher percentage of the total population that is foreign-born (0.83; 0.82–0.84). In terms of health behavior and risk factor variables, higher proportions of smokers were associated with increased mortality rates (1.05; 1.04–1.05) whereas higher proportions of obese individuals in the population was less predictive (1.00; 0.99–1.00).

The county-level characteristics and the county’s metropolitan status explain a large amount of the variation by state. As illustrated in Fig. 4, the state by year interactions in Model 3 that controlled only for year, age, sex, state and state-year interaction terms were substantially reduced in Model 4 with the inclusion of the county-level characteristics and metropolitan status. In Model 3, the mortality risk ratios relative to New York ranged from 1.02 for New Jersey to 1.52 for West Virginia. In Model 4, in which we also adjusted for metropolitan status and county-level characteristics, the risk ratios were reduced to a range of − 0.93 for New Jersey to 1.16 for Kentucky (Fig. 4). The county-level characteristics also explain variation by metropolitan status. In Model 2, mortality risk ratios for the metropolitan category by year interactions range from 1.11 for large fringe metros to 1.30 for nonmetropolitan areas relative to large central metros; these were reduced to 1.06 for large fringe metros to 1.12 for nonmetropolitan areas in the fully adjusted Model 4.

### 4.3 Results of Decomposition Analyses

**Fig. 5:**
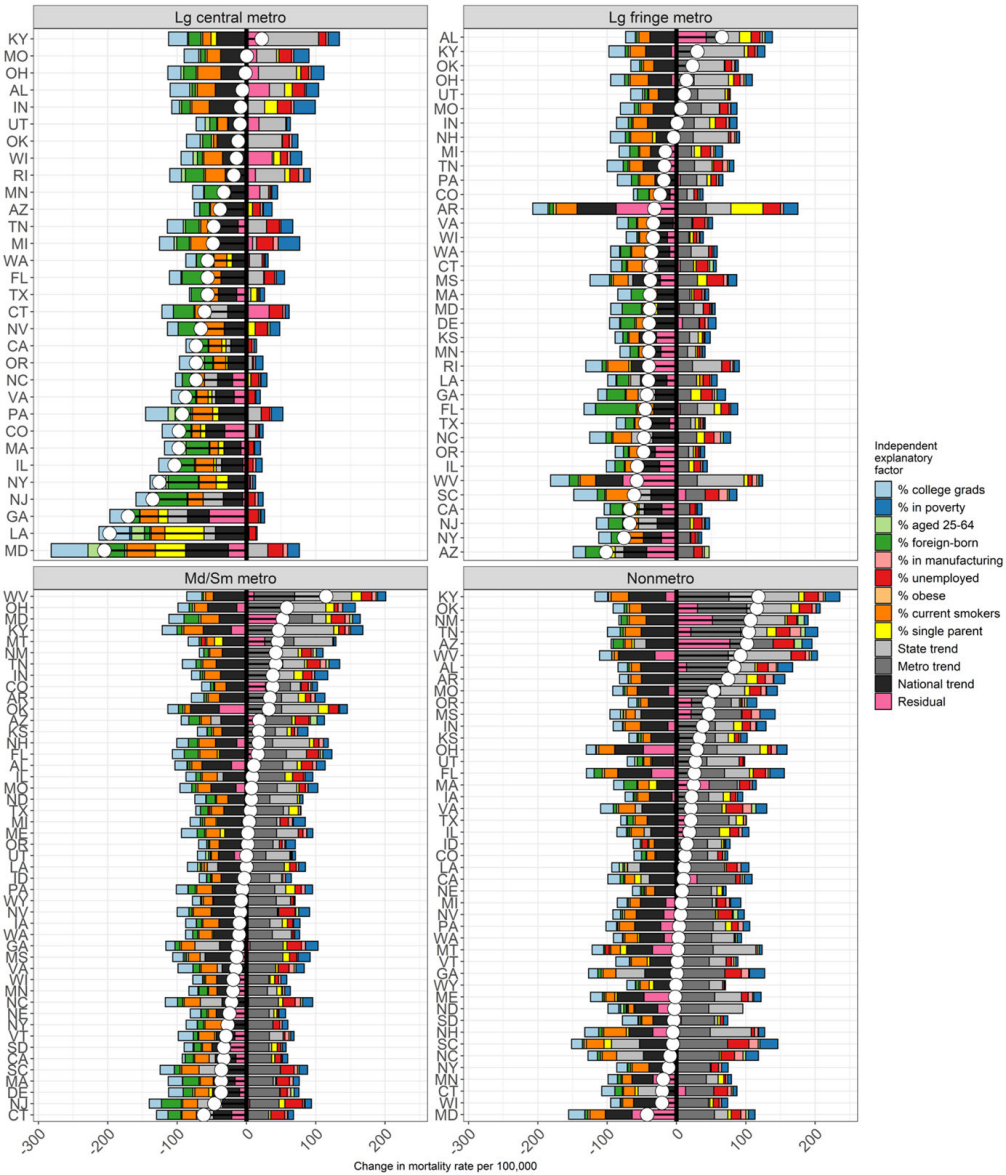
The contributions of county-level characteristics to the change in U.S. age-standardized all-cause mortality per 100,000 population between 1999–2000 and 2015–2017 by metropolitan–nonmetropolitan category and state, ages 25–64.

**Fig. 6:**
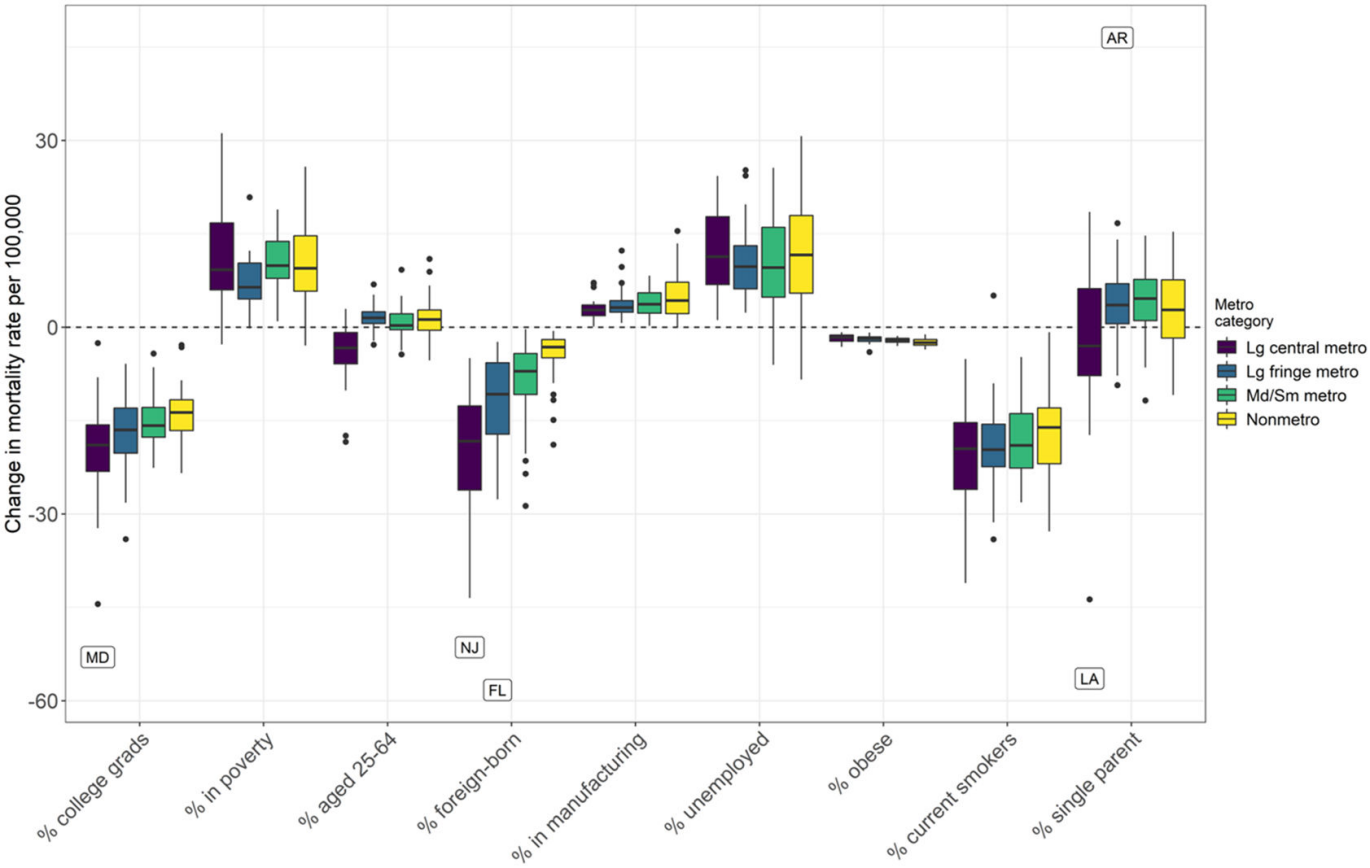
The contributions of county-level characteristics to the change in age-standardized all-cause mortality rates (ages 25–64) per 100,000 population by metropolitan–nonmetropolitan category and state between 1999–2001 and 2015–2017.

The above analyses model how county-level characteristics are associated with mortality across the entire period 1999–2001 to 2015–2017 and to what extent they explain mortality variation by state and metropolitan status. However, these conditional coefficients alone do not shed light on how much change in observed mortality over time is attributable to each time-varying county-level characteristic in our model, which depends not only on the coefficients but also the shifting distributions of these characteristics between 2000 and 2017. County-level characteristics that exhibit small or no changes between 2000 and 2017 are unlikely to explain the observed divergent mortality trends across the United States since 1999–2001, whereas other characteristics, such as obesity, smoking, and percent foreign-born are expected to make substantial contributions as their distributions have shifted more dramatically (Table 1). Table 3 and Figs. 5 and 6 present the results from the Shapley decomposition. Decomposition of the change between 1999–2001 and 2015–2017 is estimated for each county (Appendix Section 1). To illustrate the impact of the changing county-level characteristics on the state and metro–nonmetro divergence in mortality we have aggregated these additive contributions to all state and metropolitan category combinations by age-standardizing with the 2000 Census population age structure and population weighting over county and sex. Contributions from the decomposition can be interpreted as a conditional expectation under our model: the change in expected mortality associated with a change in the given time-varying county-level characteristic, compared to expected mortality if that characteristic had not changed since 2000. For additional details on decomposition, see Appendix Section 1.

Table 3 presents the contributions of county-level characteristics to the change in ASCDR per 100,000 population by metropolitan status. County-level characteristics that contributed to decline in mortality, e.g., increases in the percent college graduates, in the percent foreign-born, and declines in smoking prevalence made the largest contributions to mortality reductions in all metropolitan status categories. At the same time, these contributions were most pronounced in large central metropolitan areas and their suburbs and least prominent in nonmetropolitan areas. The largest difference was in the contribution of the percentage foreign born, ranging from a high of − 23.4 per 100,000 in large central metros to − 3.7 per 100,000 in nonmetropolitan areas. There was much less variation in the contributions of college graduates and declines in smoking prevalence. The favorable contributions were offset by increases in poverty, unemployment, single-parent households, and declines in manufacturing employment, with the largest contribution made by increases in the percent of the population in poverty and the unemployed in nonmetropolitan areas, closely followed by small/medium metros.

Figure 5 plots the contributions of the county-level characteristics by metropolitan status and state. In this plot, we also include the contributions of the average state and metropolitan category trends as well as the contribution of the national trend. Last, the contribution of the model residual represents unexplained change. With the inclusion of the contribution from the model residual, all decomposed contributions will sum approximately to the observed mortality change.

There are several features of this decomposition that stand out in Figs. 5 and 6. As previously noted, relative to large central metropolitan areas, trends by metropolitan category are important in explaining county-level differences in ASCDR trends at working-ages since 1999–2001, with nonmetro counties on average experiencing largest increases (roughly + 50 per 100 k) in mortality rates. At the same time, there is considerable variation in mortality trends by metropolitan status across and within states, as well as in the contributions of county-level characteristics by metropolitan status. For example, while increases in college attainment made positive contributions to declines in mortality everywhere, its contribution varied across states and counties with the largest contribution in large central metros recorded in Maryland (− 53 per 100,000) and the smallest in Texas (− 3 per 100,000). Similar variability is evident across states for nonmetropolitan areas with the contributions of percent college graduates ranging from a high of − 23 per 100,000 in Maryland to − 3 per 100,000 in Arizona and New Mexico. Increases in the proportion foreign-born made the largest average contribution in large fringe metros in Florida (− 58 per 100,000) and the smallest in nonmetropolitan areas of several states (− 1 per 100,000) (Fig. 5 and Appendix Tables 5 and 8). There was also variability across states in the contribution of declines in smoking prevalence by metropolitan status. For example, these contributions ranged in large central metropolitan areas from − 5 per 100,000 in Oklahoma to − 41 per 100,000 in Maryland. Similarly, there was large variation across states in nonmetropolitan areas with smallest contribution recorded in nonmetropolitan areas in Louisiana (− 1 per 100,000) and largest in New Hampshire (− 33 per 100,000) (see Appendix Tables 5, 6, 7, 8).

Increases in the percent of the population unemployed, decreases in the percent of the labor force in manufacturing, and an increase in single parent households had an offsetting impact as they contributed to increases in working-age mortality over the period studied. For example, in nonmetro counties in Tennessee the average contribution of decline in manufacturing employment was + 15 per 100,000. Proportions in poverty generally increased across all metropolitan categories but were most pronounced in contributing to mortality increases in large central metropolitan counties in Indiana and Michigan (+ 31 per 100,000). Increases in unemployment predicted increased in mortality trends across almost all counties regardless of metropolitan status (Fig. 6). This included high contributions in nonmetro counties across South Carolina (+ 31 per 100,000) and Arizona (+ 30 per 100,000), but also large central metropolitan counties in Michigan (+ 24 per 100,000) and Maryland (+ 22 per 100,000). Increases in the percent single-parent families also predicted increases in mortality, though to a lesser degree. In addition, the widespread increase in obesity was associated with increase in working-age mortality everywhere, but its contribution was small relative to the other county-level characteristics (Figs. 5 and 6 and Appendix Tables 5, 6, 7, 8).

## 5 Discussion

In recent years, adverse mortality trends at working-ages and especially in mid-life have been the focus of public attention (Case & Deaton, 2015, 2020). At the same time, there has been a growing divergence in mortality by region of the country, state, and metropolitan status (Cosby et al., 2008; Cossman et al., 2010; Elo et al., 2019; James, 2014; James & Cossman, 2017; National Academies of Sciences, Engineering, and Medicine, 2021). We confirm what others have previously documented—increasing geographic divergence in mortality by state and metropolitan status. We extend this line of research by identifying several county-level structural and population characteristics that predict changes in mortality at ages 25–64 between 1999–2000 and 2015–2017, while at the same time accounting for state and metropolitan–nonmetropolitan mortality trends and the spatial structure of county-level mortality estimates.

There are several important reasons which call for the examination of midlife mortality trends at the county-level. Spatial autocorrelation in the county-level residuals of our non-spatial models demonstrates that broad geographic groupings, such as metro–nonmetro area and state are not sufficient to capture the increasingly fractal geography of mortality in the United States. By decomposing change in the multivariate spatial model, we are able to account for spatial autocorrelation in county-level mortality while examining contributions of changing county-level characteristics to trends in working-age mortality that work in opposite directions.

### 5.1 Trends by State and Metropolitan Status

Mortality trends varied considerably by state as we have shown in Fig. 4. Compared to New York, all states experienced worse trends in working-age-mortality with 10 worst performing states located wholly or partly in Appalachia (Kentucky, Ohio, West Virginia), the South (Alabama, Arkansas, and Oklahoma), and the Mountain division (Montana and New Mexico). In addition, Indiana and New Hampshire were among the 10 worst performing states. Several of these states experienced some of the highest increases in mortality from drug poisoning (e.g., Kentucky, New Hampshire, Ohio and West Virginia) and relatively large increases in suicide mortality (Arkansas, Montana, Oklahoma), causes of death that have been identified as two key drivers of increasing working-age mortality between 1990 and 2017 (National Academies of Sciences, Engineering, and Medicine, 2021).

At the same time, we documented considerable heterogeneity in mortality trends by metropolitan status across and within states (Fig. 3). In general, large central metropolitan areas fared the best in all states with nonmetropolitan areas fairing the worst. In most states working-age mortality in nonmetropolitan areas remained stagnant or was increasing between 1999–2001 and 2015–2017. We further concluded that controls metropolitan status explained more of the county-level trends in mortality than accounting for state trends alone. This may not be surprising due to the fact that there is considerable variation in mortality across counties within states, which is clearly shown in Fig. 2. Vierboom and Preston (2020; Abstract), studying geographic divergence in mortality above age 65, similarly concluded that “metropolitan status rather region was a better predictor of mortality changes than geographic region.”

Within- and between-state differences in social, economic, health care, and policy environment are likely to play a role in explaining across and within state variation in working-age mortality. In this paper, we examine whether changes in county-level characteristics, which have been associated with working-age mortality in prior studies and which exhibited considerable change over time, can help explain trends in working-age mortality by state and metropolitan status (Dwyer-Lindgren et al., 2016; Elo et al., 2019; Vierboom et al., 2019). In addition, we include state-by-year fixed effects to account for all time-varying changes at the state-level. When county-level characteristics are controlled for in the model that also adjusts for state and metropolitan status, we explain a large fraction, although not all, of the state-level variation in mortality trends as is shown in Fig. 4. The remaining residual state-level variation may be driven by institutional arrangements at various policy levels, including state, county, and local jurisdictions. A limitation of the present study is that we do not consider the effects of specific state-level policies, but rather decompose mortality trends across various geographic levels within states. The results of our analysis highlight the difficulty of evaluating the influence of state-level policies on mortality trends given the vast heterogeneity in these trends across county- and metropolitan status within states. For example, a state-level policy may have a mean effect on all counties within the state or might only affect some counties or differentially influence counties depending on their metropolitan status, sociodemographic, or labor market profiles. It will be productive for future research on the influence of policies at various institutional levels to attend to the role of heterogeneity across the vastly different mortality profiles of counties within states.

### 5.2 Change in County-Level Socioeconomic, Labor Market and Family Characteristics

The great recession and its aftermath have drawn attention to the potential contribution of adverse economic conditions to trends in working-age mortality in recent decades (Chetty et al., 2016b). We examined the contribution of four county-level SES and labor market indicators, namely percent college graduates, percent in poverty, percent unemployed, and percent in manufacturing. In addition, we included a measure of changing family characteristics, namely percent single parent families. These county-level characteristics predicted much of the metropolitan status and state-level variation in mortality at ages 25–64 over and above national trends and state fixed effects. Declines in manufacturing employment and increases in unemployment, poverty and single parent families contributed to increases in working-age mortality. Declines in manufacturing employment were widespread and tended to be worse in nonmetropolitan areas. They made the largest contributions to the adverse mortality trends in the nonmetropolitan South. The changing educational composition at the county-level, measured by percent college graduates, in turn predicted mortality decline, and tended to favor larger metropolitan areas where the increase in the percent college graduates was greater. The one county-level SES characteristic that increased everywhere was percent poverty, with the largest increases in large central metros and in some small/medium metros. Our measure of poverty does not account for income from transfers or cost-of-living differentials, and therefore does not capture well metro–nonmetro differences in income insecurity (Nolan et al., 2017). Nevertheless, our results taken together suggest that declines in manufacturing, increases in unemployment, poverty and single parent families offset mortality improvements everywhere and that they were substantial enough to increase mortality rates on average in many nonmetropolitan areas in over half of the states.

### 5.3 Increase in the Foreign-Born Population

Increases in the foreign-born population made large contributions to the decline in working-age mortality in large metropolitan areas and in many of their suburbs. There are three reasons why the changing percentage of a county’s population that is foreign-born may be such a strong correlate of mortality improvement. First is the direct compositional effect: foreign-born have lower mortality rates, such that as the percent of the foreign-bon population increases the average population mortality rate decreases (Blue & Fenelon, 2011; Hamilton, 2020; Singh & Siahpush, 2002). The second explanation is a potential indirect compositional effect: the proportion foreign-born has some effect on increasing other unobserved characteristics of the county that are correlated with improved mortality. Third, there is the selection effect: the foreign-born population selects into counties for unobserved reasons that are correlated with lower and improving mortality rates.

The current analysis cannot tell us the extent to which each of these three components contributes to the foreign-born mortality association we observe. Future research can be useful in disentangling the different pathways through which a changing foreign-born population affects the geography of midlife mortality in the United States. However, our study does demonstrate the importance of this indicator in explaining mortality differentials at the county-level conditional on many other structural and population characteristics. Our study demonstrates that nativity is a critical dimension of mortality change and is likely to grow in importance as the foreign-born population increases (especially among those racialized as Black, a foreign-born population that is increasing rapidly and has dramatically different mortality rates than the native-born Black population) (Anderson, 2015; Hamilton, 2019). Studies failing to account for nativity might risk misinterpreting total mortality changes as true improvements in underlying mortality rates rather than a shifting population nativity composition.

### 5.4 Health-Related Behaviors

The reductions in smoking prevalence contributed to mortality decline between 2000 and 2017. The last several decades have witnessed a reduction in smoking prevalence in the United States. By 2016, only 17.5% of the men and 13.5% of the women were current cigarette smokers (Jamal et al., 2018). However, the reduction in smoking over the last decades has not been uniform across population subgroups or regions of the country. Individuals with low levels of schooling are more likely to smoke than others and people in the Midwest and the South are more likely to smoke than those who live in the Northeast or the West (Ibid). Between 1965 and 2004, smoking explained a large fraction (up to over 70%) of the growing gap in male mortality between the worst performing Census division, East South-Central consisting of Alabama, Kentucky, Mississippi, and Tennessee, and other U.S. Census divisions, and up to over 50% of the growing gap in female mortality at ages 50 and above (Fenelon, 2013). Individuals living in rural areas are also more likely to smoke than residents of urban areas, and these differences have widened over time (Doogan et al., 2017). We find that changing smoking prevalence was associated with mortality decline in all states and metropolitan status categories, although these reductions tended to be somewhat larger in large metropolitan areas. Further reductions in smoking have the potential not only to reduce working-age mortality but also narrow geographic mortality disparities.

The rise in obesity in the United States in the last several decades has been a subject of numerous studies and the upswing in obesity has been implicated in the slow U.S. mortality decline. A recent study documented that between 1988 and 2011 increasing body mass index (BMI) slowed down the annual rate of decline in U.S. death rates that is equal to a 23% relative reduction in the rate of mortality decline (Preston et al., 2018). Obesity is widespread throughout the South, especially in nonmetropolitan areas (Michimi & Wimberly, 2010). Our results reinforce the findings by Preston and colleagues (Preston et al., 2018), as we also find that the increase in county-level prevalence of obesity between 2000 and 2017 contributed to the slowdown in mortality improvements everywhere, although its impact was relatively small in the fully adjusted Model 4. Thus, the contribution of rise in the prevalence of obesity among Americans has had a widespread impact and it has contributed to the slowdown in U.S. working-age mortality.

## 6 Limitations

A clear limitation of this analysis is the associational nature of the documented relationships between working-age mortality and county-level characteristics. We can make a strong case that we have accounted for time-invariant unmeasured confounders at the state- and metropolitan-levels. However, this analysis is ultimately ecological and can therefore not be used to make causal claims. Despite this limitation, we believe the results make a strong case as to which compositional and structural shifts in counties since 2000 have likely contributed positively or negatively to trends in midlife mortality and its growing geographic divergence.

## 7 Conclusions

Divergence in mortality in young adulthood and midlife by state and metropolitan status has likely been a contributing factor to United States’ comparatively low standing internationally. Understanding the mortality trends described in this paper requires examining simultaneous divergences within the United States of economic hardship, health behaviors, and population composition not only at the state-level but also at the county-level and across metropolitan status. We found that the inclusion of metropolitan status by year interaction terms explained more of the variance than the inclusion of state by year interaction terms (Table 2, lower DIC in Model 2 than Model 3), suggesting that metropolitan status was more important in predicting working-age-mortality than state alone.

A key finding relates to the important role the increase of the foreign-born population has played in U.S. mortality trends and points to the importance for distinguishing between U.S.-born and foreign-born residents in studies of space-specific mortality trends. Another key insight gained from these analyses is the offsetting influences of changing county-level characteristics. For example, we find that expected improvements in working-age mortality associated with reductions in smoking and increases in college attainment are often entirely offset by increases in unemployment and poverty and declines in manufacturing employment. Overall, the adverse trends in the county-level characteristics outweigh the positive ones in most nonmetropolitan counties and in several small/medium metros. Only in large central metros and large metro suburbs did the positive changes outweigh the negative in many states. Given the patterns that we have observed, other unobserved factors that have contributed to the mortality divergence are also likely to vary by metropolitan status and region of the country, as is suggested by the contribution of the residual trend in the decomposition. Although we cannot establish the causality of these relationships, this study points to the importance of considering the many nuanced structural and compositional factors that are working simultaneously, and often in opposite directions, to produce subnational working-age mortality trends. Our decomposition approach helps to identify the relative magnitudes of what might be driving startling working-age mortality increases and clarifies where mortality improvements could be much faster if not for counteracting forces. Policy evaluation and action in response to mortality changes must consider these structural and contextual dynamics in order to establish holistic intervention strategies.

## Figures and Tables

**Table 1:** Age-standardized mortality rates at ages 25–64 per 100,000 population and county-level characteristics by metropolitan status in 1999–2001 and 2015–2017 (weighted by population). *Source*: For list of sources see Appendix Table 4

	Large central metro		Large fringe metro		Medium/Small metro		Nonmetropolitan	
			
	2000	2017	2000	2017	2000	2017	2000	2017
Mortality per 100 k	410.84	335.16	342.94	312.84	394.35	398.52	427.72	463.28
% College grads	28.53	33.71	28.47	33.73	22.58	26.77	15.12	18.65
% In poverty	12.49	15.95	7.31	10.35	11.70	15.77	13.87	17.35
% In manufacturing	12.17	8.89	13.44	10.19	14.65	10.95	18.68	14.08
% 25–64 year-olds	54.73	55.85	54.40	53.44	51.91	51.43	51.27	50.88
% Foreign-born	8.20	11.42	4.84	7.51	2.27	3.41	0.92	1.32
% Unemployment	4.16	5.30	3.36	4.91	4.19	5.59	4.69	5.83
% Obese	25.88	36.18	25.37	37.48	27.55	40.04	29.72	42.46
% Current smokers	23.48	16.95	23.97	17.95	25.80	20.71	28.13	24.34
% Single-parent	0.12	0.12	0.08	0.09	0.10	0.11	0.09	0.09

**Table 2:** Estimated coefficients (mortality risk ratios) for all-cause U.S. mortality, ages 25–64, 1999–2001 and 2015–2017

	Model 1		Model 2		Model 3		Model 4	
	Coef	95% CI	Coef	95% CI	Coef	95% CI	Coef	95% CI
Intercept	0.00	(0.00–0.00)	0.00	(0.00–0.00)	0.00	(0.00–0.00)	0.00	(0.00–0.00)
Female	0.59	(0.58–0.59)	0.59	(0.58–0.59)	0.59	(0.58–0.59)	0.61	(0.60–0.61)
2017	0.95	(0.94–0.95)	0.83	(0.82–0.83)	0.78	(0.77–0.79)	0.93	(0.88–0.90)
Age 30–34	1.21	(1.20–1.22)	1.21	(1.20–1.22)	1.21	(1.20–1.22)	1.21	(1.20–1.22)
Age 35–39	1.62	(1.61–1.63)	1.62	(1.61–1.63)	1.62	(1.61–1.63)	1.62	(1.61–1.63)
Age 40–44	2.28	(2.26–2.30)	2.28	(2.26–2.30)	2.28	(2.26–2.30)	2.28	(2.26–2.30)
Age 45–49	3.36	(3.33–3.38)	3.36	(3.34–3.39)	3.36	(3.34–3.38)	3.36	(3.34–3.39)
Age 50–54	5.03	(4.99–5.06)	5.03	(5.00–5.07)	5.03	(5.00–5.07)	5.03	(5.00–5.07)
Age 55–59	7.71	(7.66–7.76)	7.72	(7.67–7.77)	7.71	(7.66–7.76)	7.72	(7.66–7.77)
Age 60–64	11.47	(11.39–11.54)	11.47	(11.40–11.55)	11.47	(11.39–11.55)	11.47	(11.38–11.54)
Lg fringe metro			0.89	(0.83–0.96)			0.83	(0.74–0.79)
Md/Sm metro			0.95	(0.89–1.01)			0.82	(0.74–0.79)
Nonmetro			0.98	(0.92–1.05)			0.82	(0.72–0.77)
Year*Lg fringe metro			1.11	(1.10–1.12)			1.06	(1.07–1.09)
Year*Md/Sm metro			1.23	(1.22–1.23)			1.10	(1.11–1.13)
Year*Nonmetro			1.30	(1.29–1.31)			1.12	(1.12–1.14)
% College grads							0.90	(0.89–0.91)
% In poverty							1.04	(1.03–1.04)
% In manufacturing							0.98	(0.97–0.99)
% Aged 25–64							0.95	(0.94–0.96)
% Foreign-born							0.83	(0.82–0.84)
% Unemployment							1.06	(1.05–1.07)
% obese							1.00	(0.99–1.00)
% Current smokers							1.05	(1.04–1.05)
% Single-parent							1.07	(1.06–1.07)
DIC	513,352	506,553	506,721	501,012				

DIC = Bayesian deviance information criteria; 95% CI = 95% credible interval

All county characteristics are in terms of standard deviations

Model 1 = age, sex, year. Model 2 = age, sex, year * metro. Model 3 = age, sex, year * state. Model 4 = age, sex, year * metro, year * state, county covariates. Omitted categories: year 2000; ages 25–29; large central metro, New York State

All models include correlated normal and spatial random effects on county following the BYM model

**Table 3:** The contributions of county-level characteristics to the change in U.S. age-standardized all-cause mortality per 100,000 population between 1999–2001 and 2015–2017 (averaged across all counties within each metropolitan category, weighting by 2017 population)

	Lg central metro	Lg fringe metro	Md/Sm metro	Nonmetro
% College grads	− 15.0	− 15.9	− 14.3	− 13.9
% In poverty	8.6	6.8	10.9	11.9
% Aged 25–64	− 2.1	1.4	0.9	1.2
% Foreign-born	− 23.4	− 18.8	− 9.2	− 3.7
% In manufacturing	2.8	3.1	4.0	6.4
% Unemployed	8.0	10.1	11.1	11.6
% Obese	− 1.4	− 1.7	− 2.1	− 2.6
% Current smokers	− 21.0	− 19.4	− 19.3	− 17.8
% Single parent	− 2.9	4.0	5.3	5.0
State trends	4.2	4.4	8.6	18.5

## Footnotes

1 Northeast: Pennsylvania – 78.2 years, New York – 80.9 years; Midwest: Ohio – 77.0 years, Minnesota – 80.9 years; Continental West: New Mexico – 77.6 years, California – 81.2 years.

## Appendix

### Section 1

The basic goal of the decomposition is to estimate the additive contributions of each time-varying term in the model to the observed change in mortality rates. For linear models, this would be straightforward to calculate via the difference in conditional expectations introduced by changing one term while holding all others at their 2000 values. However, as explained below, this results in estimates that do not sum to the total observed change for non-linear models. Given our model indexed by county (*i*), year (*y*), age (*a*), and sex (*s*), *observed change between two years* for any mortality rate (*m_i,a,s_*) is a function of the following time-varying (*y*) terms: county-level covariates, ***X_i,y_***, time trends and interactions with state and metropolitan category, ***β***_2_*Y* * (***T_i_*** + ***M_i_***), and unexplained change in the residual (*ε_i,y,a,s_*).

As we include nine county-level covariates in the full model, this means there are 13 terms (including the national, state, and metropolitan trends and the residual trend) that change values between 2000 and 2017 for any given county. The Shapley decomposition estimates the *additive contribution in normal space* of a given time-varying term (*Z*) by using the fitted model to enumerate all possible permutations where that term’s value changes and all other time-varying terms (***X***) take on either their 2000 or 2017 values. For example, to estimate the contribution of changes in poverty rates, consider the set of possible permutations (*P*) of all 12 other time-varying terms, equal to 2^12^. Under each permutation, we calculate the conditional expectation of the difference in mortality rates given the value of *Z* in 2017 compared to its value in 2000 (Eq. 5). The average of these expectations over all permutations of the other time-varying terms (***X_p_***) should approximate very closely the contribution of changes in *Z* (*C_Z_*) to observed changes in mortality rates under our model.

(5)
$$C_{Z}=\frac{1}{P}\sum_{p=1}^{P}E\left[m_{i,y,a,s}|Z=Z_{2017},X=X_{p}\right]-E\left[m_{i,y,a.s}|Z=Z_{2000},X=X_{p}\right]$$

*C*_*Z*_ can be interpreted as the expected change in mortality (1999–2001 to 2015–2017) associated with changes in *Z*, compared to if *Z* had maintained its 2000 values. This provides an exact calculation of the additive *C*_*Z*_ for linear models, and the sum of all contributions will add up exactly to the total observed change. The permutation-based Shapley approach would be unnecessary in this case, as the conditional expectation of the difference would be identical under all permutations. One could simply calculate the change once while holding all other time-varying terms (***X_p_***) at their 2000 values. However, the Shapley approach can usefully provide an approximation of contributions for any non-linear model. Here, we have a logit link function, so differences under each permutation are identical in logit space but not in normal space. By averaging over normal space differences in all permutations, we are essentially averaging over non-linearity that occurs with changes at different locations in the logit distribution. Without taking this step, all contributions would not sum to the actual observed mortality change.

As a means of testing how precise the Shapley approximation is in a non-linear setting, the sum of contributions from all 13 time-varying terms should approximate very closely the actual observed mortality change from 1999–2001 to 2015–2017. We test the concordance between the observed mortality changes 1999–2001 to 2015–2017 and the sum of the decomposed changes attributable to each independent factor $\left(\sum_{z=1}^{13}C_{Z}\right)$. We aggregate to the metro-state-level via age-standardizing to the 2000 Census population age structure and population-weighting. We find error introduced by the Shapley approximation to be low and unbiased (comparison below for all-cause mortality decomposition; *R*^2^ = 0.99, mean absolute error = 2.37 per 100,000).

**Table 4:** County-level contextual characteristics by source and year coverage

	Source	Years	Extrapolated
Population with a college degree, %	2000 Census, 2014–2018 five-year pooled ACS	2000, 2014–2018	
Families with a child < 18 and single householder, %	2000 Census, 2014–2018 five-year pooled ACS	2000, 2014–2018	
Population below federal poverty threshold, %	Small Area Income and Poverty Estimates (SAIPE) Program	2000, 2015	
Proportion of employed in manufacturing sector, %	Bureau of Economic Analysis (BEA)	2000, 2017	
Proportion of labor force that is unemployed, %	Bureau of Economic Analysis (BEA)	2000, 2017	
Proportion of total population in working-ages, %	2000 Census, 2014–2018 five-year pooled ACS	2000, 2014–2018	
Proportion of total population foreign-born, %	2000 Census, 2014–2018 five-year pooled ACS	2000, 2014–2018	
Smoking prevalence (cigarette smoking in adults aged 20 and older), %	Institute for Health Metrics and Evaluation (IHME)	2000–2012	X
Obesity prevalence (proportion of adults aged 20 and older that report a BMI of 30 or more), %	Institute for Health Metrics and Evaluation (IHME)	2004–2013	X

Note: Several indicators have to be interpolated to our modelling range (2000–2017) by using linear projection with an annualized growth rate forward to 2017 or backward to 2000

**Table 5:** The contributions of county-level characteristics to the change in U.S. age-standardized all-cause mortality per 100,000 population between 1999–2001 and 2015–2017 by state, ages 25–64, large central metropolitan areas

State	Total change	% College grads	% In poverty	% Aged 25–64	% Foreign- born	% In manufacturing	% Unemployed	% Obese	% Current smokers	% Single parents	State trend	Metro trend	National trend	Residual trend
MD	−205	−53	17	−17	−36	6	22	−3	−41	−44	31	0	−62	−26
LA	−197	−44	−3	−18	−8	2	10	−2	−20	−56	−16	0	−45	4
GA	−171	−22	5	1	−21	2	18	−1	−27	−14	−28	0	−31	−54
NJ	−135	−18	7	−4	−51	4	11	−1	−22	1	−29	0	−28	−5
NY	−126	−23	4	−3	−44	3	1	−1	−24	−17	0	0	−26	5
IL	−104	−23	9	−5	−24	4	10	−2	−26	−4	−8	0	−31	−5
MA	−98	−24	9	−7	−33	1	8	−1	−12	−8	3	0	−25	−8
CO	−97	−23	5	−6	−13	1	4	−1	−12	−7	13	0	−27	−31
PA	−93	−32	17	−10	−23	2	12	−2	−29	−9	19	0	−40	2
VA	−88	−18	6	−4	−13	1	13	−2	−16	−5	−6	0	−27	−18
NC	−73	−10	8	1	−13	2	12	−1	−18	6	−19	0	−21	−20
OR	−73	−30	11	−6	−12	3	2	−1	−18	−3	8	0	−27	1
CA	−73	−10	4	−1	−21	3	6	−1	−19	−6	−7	0	−23	2
NV	−66	−15	14	3	−26	1	18	−1	−39	10	0	0	−32	2
CT	−60	−16	6	0	−30	3	20	−2	−18	−4	−25	0	−27	33
TX	−56	−3	7	0	−18	2	2	−2	−19	9	6	0	−26	−14
FL	−56	−17	12	−1	−31	2	15	−2	−24	1	25	0	−34	−3
WA	−56	−15	4	0	−24	2	3	−1	−18	−8	18	0	−20	4
MI	−48	−21	31	−4	−18	7	24	−2	−25	−14	6	0	−42	9
TN	−47	−23	17	−3	−20	2	18	−2	−10	−4	29	0	−39	−12
AZ	−38	−8	13	1	−14	3	11	−2	−11	9	−10	0	−27	−4
MN	−33	−16	8	−1	−26	2	3	−1	−9	−2	13	0	−23	19
RI	−18	−17	10	−6	−27	7	13	−2	−29	7	42	0	−31	13
WI	−14	−17	17	−6	−7	4	10	−2	−27	10	2	0	−34	37
OK	−12	−20	9	−4	−13	4	10	−3	−5	1	51	0	−40	−2
UT	−9	−13	5	−7	−10	0	1	−1	−14	−1	39	0	−26	18
IN	−9	−11	31	−3	−13	4	20	−2	−25	19	26	0	−39	−15
AL	−6	−28	18	−3	−5	2	18	−3	−26	11	22	0	−45	33
OH	−1	−19	19	−4	−18	3	11	−2	−34	6	55	0	−37	17
MO	0	−21	22	−3	−7	3	18	−3	−20	3	29	0	−37	15
KY	22	−27	16	−2	−18	3	11	−3	−12	−8	81	0	−43	23

Note: As these are additive contributions from decomposing changes to each time-varying term in a non-linear model, the state, metro, and national contributions may vary across categories (see Appendix for more information on this additive decomposition)

**Table 6:** The contributions of county-level characteristics to the change in U.S. age-standardized all-cause mortality per 100,000 population between 1999–2001 and 2015–2017 by state, ages 25–64, large fringe metropolitan areas

State	Total change	% College grads	% In poverty	% Aged 25–64	% Foreign- born	% In manufacturing	% Unemployed	% Obese	% Current smokers	% Single parents	State trend	Metro trend	National trend	Residual trend
AZ	−102	−18	0	7	−22	3	13	−2	−9	−9	−13	25	−33	−43
NY	−76	−18	4	2	−20	2	7	−2	−25	1	0	20	−26	−22
NJ	−68	−17	6	2	−21	3	12	−1	−21	5	−27	20	−26	−2
CA	−67	−9	7	1	−18	2	8	−1	−20	−2	−8	19	−25	−21
SC	−61	−34	11	2	−11	12	20	−2	−34	−2	−29	28	−37	13
WV	−57	−27	5	3	−13	7	6	−3	−22	6	68	30	−39	−77
IL	−56	−12	6	1	−13	3	10	−2	−20	7	−6	18	−24	−24
OR	−48	−12	6	1	−7	2	5	−2	−14	3	7	18	−23	−31
NC	−47	−23	11	4	−8	10	14	−2	−27	11	−30	26	−34	3
TX	−45	−13	2	2	−12	3	3	−2	−23	5	7	21	−27	−10
FL	−45	−16	10	0	−58	2	12	−2	−23	10	24	25	−33	5
GA	−43	−13	12	4	−27	3	17	−1	−17	14	−25	21	−28	−3
LA	−41	−12	8	−1	−17	2	10	−3	5	4	−14	29	−38	−14
RI	−40	−24	7	2	−6	4	12	−2	−30	−3	42	23	−30	−35
MN	−40	−14	4	1	−12	2	4	−1	−10	3	12	16	−21	−23
KS	−40	−11	8	0	−7	1	3	−2	−15	6	13	19	−25	−29
MD	−39	−16	4	2	−28	2	9	−2	−17	−4	14	21	−27	4
DE	−39	−15	11	−2	−20	5	8	−2	−22	−4	1	24	−32	9
MA	−38	−19	5	1	−26	3	12	−2	−10	3	3	19	−25	−3
MS	−38	−28	12	1	−3	5	24	−3	−22	13	−7	30	−40	−23
CT	−37	−16	4	5	−7	3	17	−2	−21	4	−24	19	−26	5
WA	−36	−13	5	−1	−14	2	5	−2	−19	−8	25	21	−28	−10
WI	−34	−15	5	1	−2	3	5	−2	−17	5	2	18	−24	−13
VA	−33	−16	6	2	−12	3	18	−2	−16	1	−6	22	−29	−5
AR	−32	−22	21	−2	−6	4	25	−4	−30	47	35	43	−56	−87
CO	−24	−6	5	3	−16	1	5	−1	−9	0	10	15	−20	−9
PA	−18	−20	6	0	−10	3	8	−2	−22	6	15	24	−31	6
TN	−17	−23	6	3	−7	7	13	−2	−20	2	25	26	−34	−14
MI	−16	−15	10	2	−13	5	14	−2	−16	10	4	22	−29	−9
NH	−4	−22	5	2	−6	6	4	−2	−31	−5	51	22	−29	2
IN	1	−17	11	1	−5	4	16	−2	−21	8	23	26	−34	−8
MO	6	−20	9	1	−6	3	12	−3	−19	1	26	25	−33	10
UT	11	−17	0	−3	−4	2	2	−2	−14	3	39	20	−26	11
OH	15	−20	9	3	−6	6	9	−2	−26	7	50	25	−33	−7
OK	23	−13	4	2	−3	4	10	−3	−15	1	42	25	−33	2
KY	30	−23	10	0	−5	3	10	−3	−22	5	70	28	−37	−7
AL	65	−14	11	4	−2	4	13	−3	−16	17	19	29	−38	43

Note: As these are additive contributions from decomposing changes to each time-varying term in a non-linear model, the state, metro, and national contributions may vary across categories (see Appendix for more information on this additive decomposition)

**Table 7:** The contributions of county-level characteristics to the change in U.S. age-standardized all-cause mortality per 100,000 population between 1999–2001 and 2015–2017 by state, ages 25–64, small/medium metropolitan areas

State	Total change	% College grads	% In poverty	% Aged 25–64	% Foreign- born	% In manufacturing	% Unemployed	% Obese	% Current smokers	% Single parents	State trend	Metro trend	National trend	Residual trend
CT	−62	−16	8	1	−19	4	20	−1	−21	3	−25	32	−26	−21
NJ	−46	−18	9	1	−29	3	26	−2	−22	8	−36	43	−35	5
DE	−37	−21	7	5	−14	5	10	−3	−20	−5	1	48	−39	−10
MA	−36	−21	9	0	−24	5	19	−2	−19	1	4	39	−31	−16
SC	−36	−20	12	2	−8	5	20	−2	−25	0	−30	47	−38	2
CA	−33	−4	8	0	−12	2	9	−2	−21	5	−9	36	−30	−15
SD	−33	−13	5	−1	−10	2	5	−2	−14	8	4	33	−27	−23
VT	−30	−22	5	2	−6	6	3	−2	−23	−2	20	33	−27	−17
NY	−27	−16	8	−1	−9	4	10	−2	−27	−1	0	38	−31	−1
NE	−25	−15	10	−1	−8	1	2	−2	−12	4	7	33	−27	−17
NC	−22	−18	14	3	−8	8	18	−2	−23	6	−31	43	−35	3
MN	−20	−13	8	−2	−12	3	1	−1	−12	7	14	30	−25	−18
WI	−19	−14	8	0	−2	3	6	−2	−16	6	2	32	−26	−17
VA	−15	−22	12	3	−10	7	16	−2	−24	3	−7	42	−34	0
MS	−14	−15	17	−2	−3	2	10	−3	−17	10	−7	53	−43	−17
GA	−13	−13	18	0	−7	6	21	−2	−20	5	−35	48	−39	5
WA	−11	−10	7	0	−7	2	3	−2	−19	−2	27	37	−30	−17
IA	−10	−15	10	0	−8	1	8	−2	−23	7	18	34	−28	−12
NV	−9	−17	16	4	−4	1	24	−2	−26	1	0	45	−36	−14
WY	−8	−12	2	−1	0	1	4	−2	−13	−1	21	43	−35	−12
PA	−6	−17	11	0	−10	5	9	−2	−22	13	16	41	−33	−16
ID	−3	−10	9	0	−6	4	0	−2	−13	2	17	33	−27	−9
LA	0	−15	10	−4	−2	3	10	−3	−5	8	−15	51	−41	4
UT	0	−12	5	−4	−3	1	1	−1	−10	1	35	28	−23	−17
OR	2	−12	14	2	−3	4	4	−2	−14	0	9	38	−31	−8
ME	2	−23	8	0	−11	5	7	−2	−20	−6	31	38	−31	5
MI	4	−15	17	1	−4	6	12	−3	−16	−2	5	44	−36	−6
TX	6	−7	1	0	−11	3	−3	−2	−18	14	8	40	−33	13
ND	7	−16	5	−1	−13	0	0	−1	−17	3	40	33	−27	1
MO	7	−17	14	0	−6	4	9	−3	−19	3	28	45	−36	−15
IL	9	−14	11	0	−7	2	16	−2	−22	11	−9	41	−33	15
AL	11	−17	13	−1	−4	6	16	−2	−15	6	21	52	−43	−22
FL	17	−17	13	4	−20	3	18	−2	−25	−3	29	48	−39	10
KS	18	−14	15	0	−6	4	5	−2	−12	6	17	42	−34	−1
NH	18	−17	7	0	−13	7	8	−2	−24	6	53	38	−31	−14
AZ	19	−11	11	9	−16	2	20	−2	−14	5	−14	45	−37	20
OK	32	−15	11	0	−6	2	14	−3	−8	15	53	51	−41	−39
AR	34	−18	14	0	−4	6	8	−2	−16	10	24	48	−39	3
CO	38	−13	8	2	−5	4	10	−1	−16	1	14	36	−29	27
IN	38	−12	19	0	−4	4	13	−2	−23	13	24	44	−36	−1
TN	42	−20	15	5	−7	8	19	−2	−19	4	32	52	−43	−1
NM	42	−6	9	5	−7	3	14	−2	−18	5	33	42	−34	−1
MT	45	−16	3	0	−3	1	−6	−2	−11	−12	56	42	−34	26
KY	46	−21	18	2	−7	6	9	−3	−28	7	76	50	−41	−22
MD	52	−22	11	4	−21	7	20	−3	−26	8	20	48	−39	47
OH	58	−16	18	0	−5	7	6	−3	−19	12	63	52	−42	−14
WV	115	−22	12	2	−2	6	15	−3	−11	14	82	59	−48	11

Note: As these are additive contributions from decomposing changes to each time-varying term in a non-linear model, the state, metro, and national contributions may vary across categories (see Appendix for more information on this additive decomposition)

**Table 8:** The contributions of county-level characteristics to the change in U.S. age-standardized all-cause 0mortality per 100,000 population between 1999–2001 and 2015–2017 by state, ages 25–64, nonmetropolitan areas

State	Total change	% College grads	% In poverty	% Aged 25–64	% Foreign- born	% In manufacturing	% Unemployed	% Obese	% Current smokers	% Single parents	State trend	Metro trend	National trend	Residual trend
MD	−42	−23	9	4	−5	7	14	−3	−22	5	19	55	−39	−64
CT	−21	−19	6	2	−11	5	20	−2	−17	−4	−27	41	−29	13
WI	−21	−14	9	0	−2	3	8	−2	−16	5	2	46	−33	−29
MN	−19	−13	6	0	−5	3	3	−2	−14	8	17	43	−30	−34
NY	−11	−15	9	−1	−4	4	11	−2	−24	1	0	49	−34	−5
NC	−9	−14	18	4	−4	12	19	−3	−21	3	−39	62	−44	−4
SC	−5	−16	26	2	−3	13	31	−3	−26	−11	−40	74	−52	−1
NH	−5	−20	9	1	−5	5	5	−2	−33	−4	59	49	−34	−34
SD	−4	−22	6	−1	−3	0	5	−2	−16	4	5	48	−34	7
ND	−3	−15	−3	−4	−4	0	−1	−2	−8	−2	49	47	−33	−27
ME	−2	−17	11	2	−3	7	10	−2	−13	−3	39	55	−39	−47
WY	−1	−12	−1	1	−4	1	2	−2	−13	−6	19	47	−33	1
GA	1	−13	22	1	−5	11	24	−3	−18	0	−41	65	−46	5
VT	1	−20	3	3	−1	4	8	−2	−23	−4	24	47	−33	−5
MT	2	−17	4	2	−2	2	−6	−2	−14	−9	62	53	−37	−35
WA	3	−13	6	3	−1	1	2	−2	−18	−3	32	51	−36	−18
PA	4	−15	10	−1	−1	7	9	−2	−22	9	18	53	−37	−23
NV	6	−11	9	7	−3	1	21	−3	−17	3	0	56	−40	−18
MI	7	−14	15	2	−2	6	13	−2	−15	2	5	52	−37	−15
NE	8	−11	4	−2	−3	1	−1	−2	−13	7	8	45	−31	6
CA	10	−18	11	3	−5	3	6	−2	−14	−9	−12	56	−39	30
LA	11	−11	11	−5	−3	6	16	−4	−1	2	−18	69	−49	−3
CO	13	−11	4	3	−6	1	7	−1	−12	−1	15	42	−29	3
ID	15	−10	6	2	−2	2	−8	−2	−8	3	20	45	−32	0
IL	19	−14	10	−2	−3	5	13	−2	−19	15	−9	53	−37	10
VA	21	−18	16	6	−5	13	24	−3	−24	6	−10	66	−46	−3
TX	21	−9	−1	1	−9	3	1	−3	−18	10	10	60	−42	16
IA	22	−12	8	−1	−3	3	8	−2	−17	10	21	46	−33	−6
MA	25	−15	6	0	−19	3	15	−1	−16	−11	3	41	−29	47
FL	26	−11	21	2	−12	4	18	−3	−19	5	36	69	−49	−36
UT	27	−11	1	−4	−8	2	6	−2	−10	−2	47	44	−31	−4
OH	29	−14	15	0	−1	8	5	−3	−23	11	62	59	−41	−48
KS	33	−12	10	−2	−4	2	7	−3	−11	9	19	52	−37	4
IN	38	−13	14	1	−1	8	12	−3	−22	12	27	56	−39	−12
OR	46	−12	14	3	−3	4	2	−3	−11	2	12	57	−40	22
MS	46	−15	22	−4	−1	12	13	−3	−13	1	−8	73	−51	21
MO	54	−15	15	1	−3	7	16	−3	−15	7	35	64	−45	−12
AR	74	−12	16	1	−1	12	12	−3	−15	15	31	70	−49	−1
AL	83	−14	22	2	−3	12	16	−3	−14	3	25	73	−52	15
WV	92	−18	9	3	−1	6	20	−3	−6	0	91	75	−53	−31
AZ	101	−3	15	11	−15	1	30	−2	−10	−3	−17	62	−44	77
TN	104	−18	18	6	−4	15	20	−3	−23	13	38	72	−51	21
NM	107	−3	6	9	−9	2	14	−3	−24	3	43	62	−44	52
OK	116	−16	6	0	−4	6	19	−3	−18	12	64	71	−50	30
KY	118	−18	21	3	−1	7	21	−3	−25	8	101	76	−53	−15

As these are additive contributions from decomposing changes to each time-varying term in a non-linear model, the state, metro, and national contributions may vary across categories (see Appendix for more information on this additive decomposition)

Another comprehensive explanation and application of the Shapley approach to national child mortality change between 1990 and 2013 is covered in Wang et al. (2014). As in their analysis, here we enumerate and average over all permutations for each of the 13 time-varying terms. It is important to note that this full enumeration would quickly become computationally burdensome with many more terms, and most likely not strictly necessary to achieve sufficient precision. If this is the case, each contribution could be approximated with a Monte Carlo approach by taking a sufficiently large random sample of all possible permutations (see Tables 4, 5, 6, 7, 8).
